# Acute Kidney Injury Pharmacokinetic Changes and Its Impact on Drug Prescription

**DOI:** 10.3390/healthcare7010010

**Published:** 2019-01-14

**Authors:** Victoria E. Blanco, Carolina V. Hernandorena, Paula Scibona, Waldo Belloso, Carlos G. Musso

**Affiliations:** 1 Human Physiology Department, Instituto Universitario del Hospital Italiano de Buenos Aires, Potosí 4234, C1199AC CABA Buenos Aires, Argentina; victoria.blanco@hospitalitaliano.org.ar (V.E.B.); carolina.hernandorena@hospitalitaliano.org.ar (C.V.H.); 2 Pharmacology Division, Internal Medicine Department, Hospital Italiano de Buenos Aires, C1199 ABH Buenos Aires, Argentina; paula.scibona@gmail.com (P.S.); waldo.belloso@hospitalitaliano.org.ar (W.B.)

**Keywords:** pharmacokinetics, acute kidney injury, drug prescription

## Abstract

Acute kidney injury (AKI) is a common problem in hospitalized patients that is associated with significant morbid-mortality. The impact of kidney disease on the excretion of drugs eliminated by glomerular filtration and tubular secretion is well established, as well as the requirement for drug dosage adjustment in impaired kidney function patients. However, since impaired kidney function is associated with decreased activity of several hepatic and gastrointestinal drug-metabolizing enzymes and transporters, drugs doses adjustment only based on kidney alteration could be insufficient in AKI. In addition, there are significant pharmacokinetics changes in protein binding, serum amino acid levels, liver, kidney, and intestinal metabolism in AKI, thus the determination of plasma drug concentrations is a very useful tool for monitoring and dose adjustment in AKI patients. In conclusion, there are many pharmacokinetics changes that should be taken into account in order to perform appropriate drug prescriptions in AKI patients.

## 1. Introduction

Acute kidney injury (AKI) consists of a fast renal function decline, which usually induces accumulation of nitrogenous waste substances in blood, and an increase in serum urea and creatinine levels. AKI is a common problem in hospitalized patients, which is associated with significant morbid-mortality, with its incidence ranging from 1–7% of all hospitalized patients to 30–50% of intensive care unit (ICU) patients [1,2]. It is worth pointing out that there has been a shift in nomenclature from “acute renal failure” to AKI, since kidney injury may have significant clinical consequences without overt renal failure [2].

Even though tubular ischaemia (oxidative stress) is an important AKI-inducing mechanism, AKI is usually multicausal, with numerous potential causes, such as sepsis, surgery, low cardiac output, hypovolemia, rhabdomyolysis, urinary obstruction, and drug toxicity [2,3].

Drug-induced AKI is a serious problem in clinical practice and accounts for around 20% cases of AKI among hospitalized patients. Usually, drug-induced AKI consists of two patterns of renal injury: acute tubular necrosis (ATN) and acute interstitial nephritis (AIN). On one hand, among the main ATN-inducing drugs are some chemotherapeutic agents (cisplatin, etc.), antibiotics (aminoglycosides, etc.), antifungal agents (amphotericin B, etc.), antivirals (foscarnet, etc.), immunosuppressive agents (cyclosporine A, etc.), bisphosphonates (pamidronate, etc.), and analgesic agents (acetaminophen, etc.). On the other hand, the main AIN-inducing drugs are some antibiotics (penicillins, etc.), anti-inflammatory agents (nonsteroidal anti-inflammatory drugs: NSAIDs, etc.), proton pump inhibitors (omeprazole, etc.), and immune checkpoint inhibitors (ipilimumab, etc.). While AIN develops from medications that incite an allergic reaction, ATN develops from direct toxicity on tubular cells [3].

Classical AKI diagnostic markers are elevated serum urea and creatinine, as well as altered urinalysis, but these markers are useless for achieving early diagnoses of AKI. Since early AKI diagnosis is crucial for taking actions in order to stop its progression and promote its recovery, novel biomarker measurement and preventive urinary indexes monitoring are the strategies that have currently been proposed to this end [1,2,3,4,5,6].

The effectiveness of a drug depends on its therapeutic level in the site of action, phenomena that usually result from an adequate balance of its administered dose, frequency of prescription, body distribution, liver metabolism, and renal clearance [7].

Acute kidney injury (AKI) is a common problem in hospitalized patients, which is associated with significant morbid-mortality. The impact of impaired kidney function on the excretion of drugs eliminated by glomerular filtration and tubular secretion is well established, as is also the requirement for drug dosage adjustment in this clinical setting [8,9].

In addition, since impaired kidney function is associated with the decreased activity of several hepatic and gastrointestinal drug-metabolizing enzymes and transporters, dose adjustments based solely on kidney alteration can be insufficient in AKI [10,11].

In critically ill patients, pharmacokinetic data is of the utmost importance to guide dosing, as these patients tend to have an increased volume of distribution, low albumin concentration, and altered drug metabolisms (hepatic, etc.) and clearances (renal, etc.) [11,12].

The main pharmacokinetic changes in critical care patients suffering from AKI are described below.

## 2. Protein Binding and Distribution

The volume of distribution for the free drug (active form) is quite different from the volume of distribution for the total drug, and the exact concentration of the free drug is extremely sensitive to plasma protein concentration and uremic toxins in AKI. In this sense, there is usually an increase in free fraction of drugs in AKI patients, since on one hand there is usually hypoalbuminemia in this setting, and on the other hand, the uremic toxins can displace drugs from their protein binding. Additionally, drug distribution depends not only on the protein binding but also on the size of the compound and nature of the tissues into which the drugs distribute; consequently, all these variables can affect the half-lives of drugs. Besides, blood flow distribution to splanchnic circulation, skeletal muscle, and fat is altered in AKI, therefore the apparent volume of distribution of drugs is also changed over the dosing cycle and illness course [9]. The free fraction of many drugs is increased in AKI, and even the volume of distribution of all the drugs can be increased because of the movement of unbound drugs into the interstitial space in this clinical setting [13].

**Figure 1:**
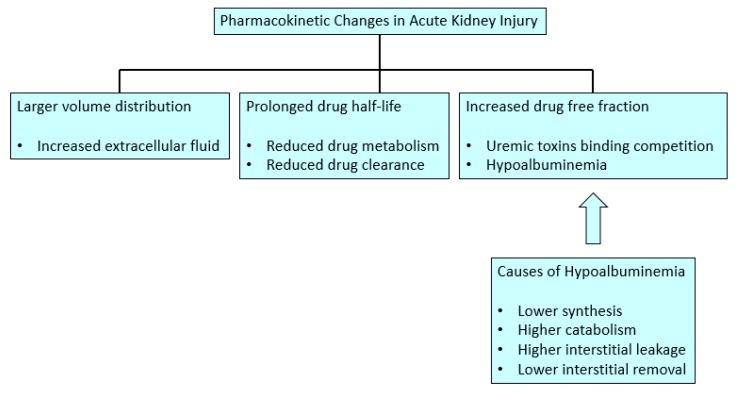
Pharmacokinetic changes in acute kidney injury.

Albumin has a shorter half-life secondary to a catabolic status in AKI, except during transcapillary albumin flow, where it shows a larger degradation rate [13]. There is an alteration in the albumin distribution in critical care AKI patients due to the capillary leakage that happens during sepsis and major surgery secondary to endothelial barrier dysfunction, which allows the proteins, inflammatory cells, and large volumes of fluid loss into the interstitial space. This volume expansion increases the volume of distribution for hydrophilic drugs. Moreover, studies demonstrate that these patients have a reduced lymph flow rate and consequently a lower serum albumin concentration [13]. Regarding albumin synthesis, it can be reduced during AKI due to a reduced gene transcription secondary to the acute phase reaction mediated by cytokines tumor necrosis factor-alpha (TNF alpha) and interleukin-6 (IL-6). In diabetic AKI patients, a low insulin activity can reduce the albumin synthesis speed, which can improve with insulin administration. Conversely, corticosteroids, steroids, and amino acid supplies can induce higher serum albumin levels because they can increase its gene transcription. However, steroids can also increase albumin catabolism. Finally, protein malnutrition has a negative impact on albumin production, as well as diets without particular amino acids (tryptophan and ornithine), which have an important effect on albumin production [3,14] (Figure 1).

Despite the fact that, in the past, treating critically ill patients with exogenous albumin to correct their hipoalbuminemia was not supposed to be beneficial, the current body of evidence indicates that hypoalbuminemia not only can contribute to the induction AKI, but also that human albumin administration has the potential to prevent AKI [15,16,17].

## 3. Amino Acids

Increased urea concentration can alter the hepatic ornithine cycle balance, and the decreased renal arginine production, which is mainly of renal synthesis, can imbalance the ornithine cycle in some AKI settings [14]. In addition, serum concentrations of amino acids, including homocysteine, dimethylarginine, and methionine, are significantly increased in AKI patients, despite no clinical liver injury. The clinical consequences of increased amino acids are unclear, but they can exacerbate metabolic acidosis [14].

It is worth mentioning that serum indoxyl sulfate (IS), a bacterial derivative of tryptophan that is >90% protein-bound, has been linked to AKI progression rate, since it stimulates inflammation, proinflammatory cytokine release, and oxidative stress in various cells, including endothelial cells and fibroblasts [14]. Hepatic function may also be compromised, as IS alters hepatic cytochrome P450 (CYP) and drug transporter function, due to IS being an agonist of the aryl hydrocarbon receptor site, which is present in the promoter of CYP and transporter genes. Furthermore, IS (and related molecules) decreases drug metabolism in hepatocytes and hepatic microsomes, but also acts as a protective agent against proinflammatory cytokine-mediated suppression of albumin release from hepatocytes in animal models [8,14].

## 4. Drug Excretion in AKI

Non-glomerular drug excretion comprises all drug removal pathways aside from the glomerular clearance. These include mainly the hepatic, pulmonary, intestinal, and even renal tubular pathways. Non-glomerular drug depuration is mainly represented by hepatic metabolism, which converts medications to less toxic and more water-soluble compounds to ease elimination from the body. Although evidence is still scarce, non-glomerular drug depuration has also been shown to be affected by AKI. Therefore, it should be taken into account that AKI changes the renal clearances (glomerular and tubular) of drugs as well as their non-renal clearances (hepatic, intestinal). This is supported by the fact that the drugs that are primarily hepatically eliminated may accumulate during AKI. Ideally, the monitoring of serum drug concentrations or pharmacodynamic responses should take place in a clinical setting, even for drugs that are considered to be predominantly hepatically cleared [10,12] (Table 1).

## 5. Hepatic Clearance in AKI

Among the relationships currently described between liver and kidney function is how AKI can affect every stage of hepatic drug metabolism, such as drug uptake (membrane transport protein function), drug modification (phase I, phase II, or both), and metabolite excretion from hepatocytes either into bile or blood [8,14,18]. Uremia and/or azotemia change hepatobiliary drug metabolism, probably through product inhibition by accumulated metabolites. However, impaired kidney function is additionally associated with decreased activity of several hepatic and gastrointestinal drug-metabolizing enzymes and transporters. CYP450 expression is also reduced in AKI [9,19].

Asymmetric dimethylarginine (ADMA) is a competitive nitrogen oxide synthase inhibitor whose clearance takes place about 80% in the liver. ADMA accumulates in AKI causing endothelial dysfunction. This suggests that hepatic function might be affected during AKI.

Furthermore, ADMA contributes to the relative intrahepatic nitric oxide (NO) deficiency typical of endothelial dysfunction. Therefore, ADMA accumulation in AKI could explain in part the hepatic dysfunction usually associated with AKI [14].

Another example of the liver–kidney crosstalk in AKI is the case of the monomethyl-aminoantipyrine (MMAAP), which is a substance mainly metabolized in the liver (90%) but whose clearance was reported to be markedly reduced in AKI. This phenomenon can be explained by the characteristic reduced hepatic metabolism already described in AKI [10,14].

The activity of cytochrome P450 (CYP) subtypes CYP3A4 and CYP3A5 has been investigated in critically ill AKI patients, by the administration of intravenous midazolam. This study has found that there was a reduction in the midazolam elimination in AKI patients, and this seems to be increased during longer periods of AKI [20]. This phenomenon has been interpreted as an altered midazolam metabolism secondary to an AKI-induced hepatic enzymes dysfunction [8,14]. Protein expression and enzyme activity data indicates that only CYP3A5 expression occurs at a level that is pharmacologically significant in this group [9].

Despite some evidence that has linked the delayed biliary excretion of some antibiotics (e.g., ceftriaxone) in post-operative pneumonia in AKI patients, it is not clear yet if this is due to sepsis rather than AKI-induced liver dysfunction [14].

Hepatic clearance (CL_H_) is the most important contributor of non-renal clearances, and it can be represented by the following Equation (20):

CL_H_ = Q_H_ × [fu × CL_int_/Q_H_ + fu × CL_int_] (1)

where Q: blood flow, CL: clearance, H: hepatic, int: intrinsic hepatic, fu: fraction of unbound drug.

The main value of this equation in clinical practice is to make physicians take into account that the CL_H_ of drugs can be reduced when patients have a reduced hepatic blood flow (e.g., sustained hypotension), reduced intrinsic hepatic metabolism (e.g., liver insufficiency), and/or a medical prescription based on high-protein binding drugs [20,21].

AKI affects every aspect of CL_H_, and CYP activity is the limiting step of the intrinsic hepatic clearance rate, which is in charge of about 50% of the drugs metabolism. Altered renal function such as AKI can modify the CYP3A function [20].

## 6. Cytochrome P450 in AKI

The cytochrome P450 (CYP) is the main enzyme system involved in drug metabolism; these enzymes are mostly abundant in the liver, hence most drug metabolism is determined by hepatic function, although it should be noted that they are significantly present in other organs such as the kidney, small intestine, lungs, and brain [8]. For instance, CYP3A4 is considered one of the most important drug-metabolizing enzymes, and it is mainly located in the liver, but the kidneys and other tissues contain this enzyme too [8]. Additionally, it should be taken into account that changes in CYP function in a particular organ, for instance in the liver, cannot be extrapolated to other organs [10].

As was mentioned above, hepatic metabolism of drugs can be altered in AKI, with these alterations based on changes in CYP enzymes transcription and translation [8,14]. In this sense, high levels of IL-6, which has an important role in AKI inflammation, reduce the inducibility of the CYP subtypes. Furthermore, the IL-6 mediates the hypothalamus–pituitary–adrenal axis activation, increasing serum cortisol concentration, which competitively inhibits the metabolism of CYP substrates [8,14]. Since IL-6 has a central role in inflammation and AKI-induced injury, anti-IL-6 monoclonal antibody could have a useful role in AKI treatment.

It has been documented that the use of the anti-IL-6 monoclonal antibody tocilizumab in AKI caused a 57% decrease in the area under the curve (AUC) of the simvastatin, which is a CYP3A4 substrate, increasing its elimination [14].

In regard to AKI etiology, it seems that it could have a different impact on other organs such as the liver. For instance, diltiazem hepatic metabolism has been documented to increase or decrease during AKI depending on its etiology. This phenomenon explains why not all CYP enzymes are affected by AKI, and their compromise may depend not only on the AKI but also on its injury mechanism [10].

Regarding the influence of dialysis on CYP3A4 activity, studies have shown that patients had a 27% increase in CYP3A4 activity 2 h after dialysis compared with the hours before dialysis, and CYP3A4 activity has been inversely related to blood urea nitrogen (BUN) concentration. In addition, the application of conventional hemodialysis in uremic state acutely improves the CYP3A4 function [10].

## 7. Renal Clearance and Glomerular Filtration Rate (GFR) Evaluation in AKI

On one hand, the kidney presents exclusive enzyme systems that have an important role in drug metabolism, as is the case of flavin-containing monooxygenase type 1 (FMO1), which participates in the oxidation of xenobiotics that contain nitrogen, sulfur, or phosphorus [8,20]. Moreover, the kidneys stand out in the metabolism of drugs, since they present highly specific enzymatic activity in particular regions, such as the CYP enzymes, which are almost exclusively found in the tubular cells within the renal cortex [8].

On the other hand, the luminal and plasma membranes of renal tubular cells present high-capacity transporters. The function of these transporters varies with the site and characteristics of the tubular epithelial cell where they are located. The drug transporters can be classified into organic anion transporters (OATs), organic cation transporters (OCTs), ABC transporter family (multidrug resistance-associated proteins family and MDR1/P-glycoprotein), breast cancer resistance protein 1, and the multidrug and toxin extrusion transporters family (MATE). The OAT families are probably one of the most important groups in terms of drug transport [7,8]. As tubular dysfunction sets in during AKI, alterations in drug transporters activity can be seen. It is thought that the OAT proteins work through a remote sensing system, as there is evidence that changes in OAT function in one organ have repercussions in other organs as well. These changes may be beneficial in AKI, as when a renal OAT system fails and there is an upregulation of OAT proteins elsewhere, that helps prevent toxin accumulation [8,10,22]. Additionally, there can also be a suppression of MDR1/P- glycoprotein activity during AKI, and this may have an impact on the clearance of drugs such as digoxin, methotrexate, and vincristine [8,10]. In addition, chronic nephropathy may decrease the excretion of drugs, which are not eliminated by glomerular clearance but by tubular drug-metabolizing enzymes and transporters [20].

Regarding GFR evaluation in AKI, estimated GFR is a less accurate means in which to guide dosing in the non-steady state than in the steady state [23]. Equations to estimate GFR in chronic kidney disease are not accurate in AKI. The Jelliffe equation seems to be the most appropriate for unstable kidney function patients, because this equation is calculated on the basis of the volume of distribution and creatinine kinetics rather than steady state parameters such as body weight or age (Table 2 and Table 3) [24,25,26,27]. This equation represents a general dynamic model of creatinine kinetics in men, and permits estimation of creatine clearance (CC) from age, body weight, the present day’s morning serum creatinine (C^1^) and the previous day’s morning serum creatinine (C^2^), when serum creatinine levels are changing. However, when serum creatinine is rising, C^2^ should be used in the equation instead of the average between C^1^ and C^2^ (Cavg), thus making the model more responsive to rapid rises in serum creatinine induced by AKI.

At present, short-timed urine creatinine clearance (CC) is the best available estimate of kidney function in AKI. However, the CC can overestimate the GFR, since creatinine is not only excreted by the glomeruli but also by the tubules [23]. It is worth mentioning that the Jelliffe GFR equation has shown a good correlation with CC in critical care patients [24,25,26,27].

## 8. Intestinal Clearance in AKI

Drug metabolism within the intestinal wall is relevant for some orally administered medications. Enteric absorption in critically ill patients is quite unpredictable for several reasons, such as changes in the gastric pH, fluid overload, and gut edema, as well of loss of enteric architecture, cholestasis in patients with septic shock, disruption of epithelial junctions, loss of enteric mucosa, and altered hepatic first-pass by systemic shunts. All these variables affect the absorption across the enteric mucosa and the bioavailability of the drugs [9,19].

Approximately 80% of the small intestine drug metabolism is performed by the CYP3A, but this amount represents only 1% of the total body’s CYP3A activity. Uptake from the intestinal lumen happens by diffusion or active OAT-mediated transport. The impact of AKI on this process remains unclear, although it is thought that intestinal motility, flora, and perfusion may be altered in critically ill AKI patients [28].

## 9. Dose Adjustment in AKI Patients—Recommendations

The determination of plasma drug concentrations is a very useful tool for monitoring and dose adjustment in patients with significant pharmacokinetic variations such as patients with AKI [14]. Any drug with low predictability between dose and effect, with a narrow therapeutic range, with non-linear pharmacokinetics, or with a significant risk of toxicity or lack of efficacy is a potential candidate for plasma monitoring [28]. In addition, close monitoring of drug therapeutic response and potential toxicity should also be performed in order to adjust the prescribed medication dose in this setting. Moreover, the prescription of nephrotoxic drugs (e.g., aminoglycosides, NSAIDs, etc.) should be avoided, if possible. However, when nephrotoxic drugs are needed for clinically compelling reasons, their nephrotoxic effect should be mitigated by avoiding concomitantly administering several nephrotoxic drugs, if/when possible [24]. The assistance of a pharmacist can be helpful in this strategy, as well as in determining dosing and dosing intervals [23].

Renin-angiotensin-aldosterone system blockers (e.g., angiotensin-converting enzyme inhibitors, angiotensin II receptor antagonists) are usually prescribed in high risk AKI individuals, such as cardiovascular, chronic kidney disease, and elderly patients. It is routinely recommended that these drugs should be discontinued during AKI, since they can contribute to exacerbating this syndrome, particularly in the setting of acute hypovolemia, despite there being little evidence to support this recommendation, and their re-prescription is usually considered when GFR has stabilized and volume status is optimized [24].

In the case of patients with antibiotic therapy, the antibiotics that can currently (and should) be monitored in critical patients are the aminoglycosides and vancomycin, which, at the risk of being at subtherapeutic levels, add the characteristic of presenting narrow therapeutic ranges. Other antimicrobial agents, such as ß-lactam antibiotics—the most commonly used group in critical care units—would also benefit if plasma levels could be monitored, given the frequency of suboptimal levels found in these patients. However, even with the availability of plasma monitoring, establishing the appropriate doses/concentrations requires more than one measurement, and their plasma measurements are not always available.

Except in cases in which a particular dose-related side effect is a known concern, it may be preferred to err on the side of higher not lower doses. Some data supports the reduction of the initial antibiotic dose only on the basis of kidney failure, when the volume of distribution of the free drug tends to cancel the effects of the hypoalbuminemia that tends to increase the free fraction of drug, whereas extracellular fluid volume expansion dilutes that free fraction more than in a normovolemic patient [28].

Regarding anticoagulant drugs, they should be used with caution in AKI due to the increased bleeding risk attributed to uremic platelet dysfunction and reduced low-molecular weight heparins and oral anticoagulants excretion [23]. Even though it has been suggested that low-molecular weight heparin dose should be determined by monitoring anti-factor X activity, many experts recommend using unfractionated heparin in severe AKI [23]. For patients with active bleeding in uremia, desmopressin and cryoprecipitate can be used to rapidly diminish bleeding time, while conjugated estrogen and dialysis without anticoagulation can be used for more prolonged bleeding control [23].

It is worth mentioning that an early AKI diagnosis is very important not only in order to perform prompt AKI treatment but also to implement an early drug dose adjustment in this setting, and consequently avoid drug toxicity. In this sense, two clinical strategies have been proposed. Firstly, AKI biomarkers measurement, which consists of a structural, physiological, biochemical, or genetic parameter change (serum or urine) that indicates the presence, severity, or progress of AKI. Kidney injury induces biological and molecular changes, which evolve to cellular damage, which can be detected early by biomarkers, since they represent the early stress response of the tubules injury. These biomarkers can detect AKI long before the rise of serum creatinine. There are different AKI biomarker types: an increased urinary excretion of proximal tubule proteins due to tubular damage (e.g., *N*-acetyl-β-d-glucosaminidase), proximal tubular dysfunction detected by decreased tubular reabsorption of filtered proteins (e.g., cystatin C), tubular genes and proteins immediately upregulated in response to injury (e.g., kidney injury molecule-1 KIM-1, neutrophil gelatinase-associated lipocalin), and urinary excretion of injury-induced mRNA levels (e.g., vanin-1, monocyte chemotactic peptide-1 (MCP-1)) [2,3,4,5,6]. Secondly, the concept of renal preventive monitoring, which is based on the idea that AKI diagnosis should be based not on monitoring a glomerular filtration rate (GFR) marker (e.g., estimated GFR based on serum creatinine) after AKI clinical diagnosis but on monitoring a urinary index (e.g., fractional excretion of sodium) before AKI installation, thereby performing preventive renal monitoring. In this sense, early AKI diagnosis is not based on looking for a particular absolute value of a urinary index but on a significant change (increase or decrease) in a urinary index value with respect to its previously known basal value [1].

## 10. Conclusions

There are many pharmacokinetics changes that should be taken into account in order to perform appropriate drug prescription in acute renal injury patients.

## Figures and Tables

**Table 1:** Drug excretion pathways.

Drug Excretion	Pathway
Renal Drug Excretion	Glomerular clearance
	Tubular clearance (secretion—metabolism)
Non-Renal Drug Excretion	Hepatic
	Intestinal
	Lung

**Table 2:** Creatinine clearance equation (CCe) (Jelliffe equation).

**V (C**^2^**− C**^1^**/T)** = Padj − Cavg × CCe ×1440
**CCe** = [−V (C^2^ − C^1^/T) + Padj]/Cavg × 1440

**V:** 0.4 × body weight (in hundreds of grams). **C**^1^**:** the present day’s morning serum creatinine (mg/mL). **C**^2^**:** the previous day’s morning serum creatinine (mg/mL). **T:** time in days between the two serum creatinine samples. **Padj:** adjusted creatinine production (mg/day). **Cavg**: average between C^1^ and C^2^.

**Table 3:** Estimated creatinine production (P).

**P** = 1344.4 − 43.76 C
**P1** = 1344.4 − 43.76 × Cavg
**P2** = 1344.4 − 43.76 × 1.1
**R** = P1/P2
**E** = 29.305 − 0.203 A (in years)
**Padj** = E × R

**P:** adjusted creatinine production. **C:** serum creatinine (mg/dL). **Cavg:** average between C^1^ and C^2^. **E:** creatinine excretion (mg/kg/day). **A:** age. **Padj:** adjusted creatinine production (mg/day).
